# Feasibility of Sodium Bicarbonate Ingestion in Artistic Swimming Performances

**DOI:** 10.3390/nu17193029

**Published:** 2025-09-23

**Authors:** Heather M. Logan Sprenger, Temisia van Biljouw, David J. Bentley

**Affiliations:** Faculty of Health Science, Ontario Tech University, Oshawa, ON L1G 0C5, Canadabentley.dj@gmail.com (D.J.B.)

**Keywords:** sodium bicarbonate (NaHCO_3_), artistic swimming, synchronized swimming, ergogenic supplementation

## Abstract

**Purpose**: We evaluated the feasibility of individualized sodium bicarbonate (NaHCO_3_) supplementation and its physiological effects on simulated artistic swimming duet performance, including blood buffering responses, perceived exertion, gastrointestinal (GI) tolerance, and performance scores. **Methods**: Seventeen (*n* = 17) elite adolescent female artistic swimmers completed an initial trial to determine individual time-to-peak blood bicarbonate concentration (Part 1). Subsequently, a subset (*n* = 7) completed a randomized, double-blind, crossover intervention (Part 2), performing competition duet routines (4 min) after ingesting either 0.3 g/kg NaHCO_3_ or a placebo timed to their individual alkalosis peak. Blood gas and lactate samples were taken pre- and post-performance. Performance was scored by blinded FINA adjudicators. GI discomfort was assessed before and after each routine. **Results**: Peak blood bicarbonate occurred at 52 ± 9 min post-ingestion, with a mean increase of 6.7 ± 1.8 mmol/L (**g** = 5.03). In Part 2 (*n* = 7), NaHCO_3_ significantly elevated pre- and post-performance pH (7.46 ± 0.02 vs. 7.37 ± 0.01; 7.34 ± 0.02 vs. 7.26 ± 0.03), HCO_3_^−^ (29.5 ± 0.9 vs. 22.4 ± 0.4 mmol/L; 21.5 ± 1.2 vs. 15.7 ± 1.5 mmol/L), and base excess (5.9 ± 0.6 vs. −2.9 ± 0.5 mmol/L; −4.3 ± 0.8 vs. −10.3 ± 1.1 mmol/L) compared to the placebo (all *p* < 0.05, **g** = 3.99–14.93). Post-performance lactate was higher (9.3 ± 1.0 vs. 8.4 ± 0.9 mmol/L, **g** = 0.89), while rating of perceived exertion (RPE) was lower (12.9 ± 0.7 vs. 14.4 ± 0.7, *p* < 0.05, **g** = −2.14). Propulsion improved (6.66 ± 0.16 vs. 6.52 ± 0.20, **g** = 0.85), with no change in execution. Mild gastrointestinal symptoms were transiently elevated with NaHCO_3_. **Conclusions**: Individualized NaHCO_3_ dosing is a feasible and effective ergogenic strategy for artistic swimmers, enhancing systemic alkalosis and perceptual tolerance while preserving technical execution. These findings support the sport-specific integration of NaHCO_3_ to optimize anaerobic performance elements in high-level artistic swimming.

## 1. Introduction

Artistic swimming is a discipline that integrates substantial anaerobic and aerobic demands with the simultaneous requirement for technical precision, prolonged apneic control, and aesthetic execution. Despite its long-standing presence at the Olympic and international level, the physiological profile of artistic swimming remains comparatively underexplored relative to other aquatic and anaerobic sports [1,2,3,4,5,6]. Approximately half of a competitive routine is performed under breath-hold conditions, resulting in repeated hypoxic exposures and significant metabolic strain [5,6,7]. Routines lasting 2.5–4 min combine high-intensity propulsion elements, static figures, and extended underwater sequences, requiring the coordinated use of both aerobic and anaerobic energy systems [5,6,7,8,9].

Although post-performance blood lactate concentrations of ~8–10 mmol·L^−1^ have been observed in elite artistic swimmers, reflecting a heavy reliance on anaerobic glycolysis, aerobic contributions are less easily quantified due to the methodological challenges of measuring VO_2_ kinetics in aquatic environments [5,6,7,8,9]. Nevertheless, recent evidence has demonstrated positive associations between VO_2_max, lactate response, and routine performance, underscoring the importance of conditioning both energy systems for optimal execution [8].

One of the most well-established ergogenic aids for exercise performed under high-intensity or hypoxic conditions is sodium bicarbonate (NaHCO_3_). Acting as an extracellular buffer, NaHCO_3_ increases blood bicarbonate (HCO_3_^−^) and pH, thereby enhancing the efflux of hydrogen ions (H^+^) and delaying acidosis-induced fatigue [3,10,11,12,13,14]. This mechanism has proven effective across a variety of intermittent and anaerobic sports, including swimming, rowing, and middle-distance running [10,12,13,14,15]. However, the ergogenic response to NaHCO_3_ ingestion is often inconsistent, in part due to inter-individual variability in the timing of peak alkalosis and the prevalence of gastrointestinal (GI) side effects [3,16].

The unique physiological demands of artistic swimming—characterized by repeated high-intensity efforts under apnea (~2 min of a 4 min routine, typically in 15–45 s bouts)—make the sport a strong candidate for NaHCO_3_ supplementation to mitigate combined metabolic and respiratory acidosis. Unlike pool swimming, where NaHCO_3_ benefits have been documented primarily during normocapnic high-intensity intervals or time-trials with only brief underwater phases, artistic swimming imposes an additional CO_2_-driven respiratory acidosis, compounding metabolic acid–base stress [4,12]. Comparisons with rowing also highlight that NaHCO_3_ appears to preferentially enhance propulsion-heavy, anaerobic tasks, though its impact under apneic constraints has not been studied [14].

Recent advances in individualized supplementation protocols, in which exercise is aligned with each athlete’s personal time-to-peak alkalosis, have improved both efficacy and GI tolerability [15,17,18,19]. Yet this approach has not been examined in artistic swimming, despite the sport’s ideal physiological profile for buffering interventions. Furthermore, adolescent female athlete who remain underrepresented in supplementation research—may particularly benefit from individualized protocols that maximize efficacy while limiting GI distress [16,20].

Accordingly, the purpose of this study was twofold: (1) to determine the individual time to peak blood bicarbonate concentration following ingestion of 0.3 g·kg^−1^ NaHCO_3_, and (2) to evaluate the physiological, perceptual, and judged performance effects of individualized NaHCO_3_ ingestion during simulated artistic swimming duet routines. We hypothesized that aligning routine performance with peak alkalosis would enhance buffering capacity, reduce perceived exertion, and selectively improve propulsion-related outcomes without compromising execution.

## 2. Methods

Seventeen (*n* = 17) competitive female artistic swimmers (mean ± SD: age 16.5 ± 1.0 y, 51.3 ± 4.2 kg) with a training age of 6 ± 2 yrs in the sport of artistic swimming and weekly training volume of 14 ± 2 h per week. All participants were members of a homogeneous national-level training group and voluntarily took part in the study. Each athlete provided both verbal and written informed consent/assent, and the protocol was approved by the Research Ethics Board of Ontario Tech University (REB#15086). None of the athletes had previously used ergogenic aids or had prior experience with sodium bicarbonate supplementation.

### 2.1. Study Design

There were two parts to the study (Part 1 and Part 2). Part 1 (*n* = 17) of the study was a single trial to determine the individual time to peak blood NaHCO_3_. Part 2 followed with a subset (*n* = 7) of the 17 athletes in Part 1, who completed a double-blind crossover intervention to evaluate the effect of the individual dose (determined in Part 1) on blood gas response, simulated duet performance, and gastrointestinal (GI) tolerability. Due to the training and competition commitments of the 17 athletes, only 7 athletes were able to commit to the completion of Part 2 during this phase of their training season—this is the rationale for the subset of athletes used in Part 2.

#### 2.1.1. Part 1: Determination of the Individual Time to Peak Blood NaHCO_3_ and Gastrointestinal Tolerability

Part 1 aimed to (1) determine the individual time to peak blood bicarbonate (alkalosis) following ingestion of 0.3 g/kg NaHCO_3_ in 17 trained artistic swimmers, to guide personalized timing for Part 2; and (2) assess gastrointestinal (GI) tolerability of the bolus dose as a familiarization trial prior to performance testing.

Seventeen elite female artistic swimmers (*n* = 17) arrived at the testing facility in a postprandial state (~4:00 PM), having consumed a standardized meal two hours prior in accordance with a trial-day nutrition plan. This nutritional protocol, including meal timing, was replicated across all experimental sessions (Parts 1 and 2). Upon arrival, participants voided their bladder and provided a midstream urine sample to assess pre-test hydration status via urine specific gravity (USG), followed by body mass measurement using a calibrated scale (SECA 874, Hamburg, Germany) to the nearest gram. Participants were classified as euhydrated if USG was <1.020. Body mass was used to calculate the individualized NaHCO_3_ dose (0.3 g/kg), a dose previously documented by Hadzic et al. (2019) in a systematic review as being an effective dosage with good gastrointestinal (GI) tolerability [13]. Under aseptic conditions, a fingertip arteriovenous blood sample (~1 mL) was collected to determine baseline blood bicarbonate (HCO_3_^−^) concentration using a handheld point-of-care analyzer (CG6+ cartridge, Abbott i-STAT 300-G, Abbott Park, IL, USA). Participants then ingested 0.3 g/kg of flavorless sodium bicarbonate powder dissolved in 300 mL of flavored water (Gatorade G2) over a 5 min period. Time zero (t = 0) was defined as the moment of full ingestion, after which blood samples were collected every 15 min at t = 30, 45, 60, 75, and 90 min, or until a peak HCO_3_^−^ concentration was observed [19].

At 15 min after ingestion and at peak alkalosis participants were asked to self-report subjective GI symptoms. Blood sampling continued until a peak in blood HCO_3_^−^ concentration was identified, followed by a subsequent decline confirmed by two consecutive arteriovenous samples. At that point, testing was concluded for Part 1. Participants then commenced their regular swim training session, serving as a familiarization opportunity for exercising under conditions of NaHCO_3_-induced alkalosis.

#### 2.1.2. Part 2: Effect of NaHCO_3_ on Simulated Artistic Swimming Performance

One week after completing Part 1, a subset of seven athletes (*n* = 7) proceeded to Part 2, a randomized, double-blind, crossover intervention. Participants completed two simulated duet competitions following ingestion of either a placebo (PL) or a 0.3 g/kg sodium bicarbonate (NaHCO_3_) solution, identical in dosing protocol to Part 1. The order of treatments and the identity of the beverages were blinded to both participants and researchers to maintain double-blind conditions. Each athlete served as her own control, completing both trials separated by a 7-day washout period. To minimize expectancy effects, participants were not informed about the true purpose of the study; instead, they were told that the investigation focused on flavored beverage preferences.

Participants reported to the testing facility at the same time of day as Part 1 (~4:00 PM), having followed the identical standardized nutrition protocol. Upon arrival, they voided their bladder and provided a midstream urine sample for the assessment of hydration status via urine specific gravity (USG). Body mass was measured using a calibrated scale (SECA 874, Hamburg, Germany) and used to calculate the individualized bolus dose of sodium bicarbonate (0.3 g/kg), consistent with Part 1 procedures. Beverage preparation—either placebo or NaHCO_3_—was conducted by a non-affiliated individual who was not involved in data collection or aware of the study hypotheses, ensuring the integrity of the double-blind design.

Following initial assessments, participants provided an arteriovenous fingertip blood sample (~1 mL) under aseptic conditions for baseline analysis of blood bicarbonate (HCO_3_^−^, mEq/L), blood glucose (mmol/L), blood gases (pO_2_, pCO_2_ [mmHg], TCO_2_ [mmol/L]), base excess (BE), and pH using a handheld point-of-care analyzer (EG6+ cartridge, Abbott i-STAT 300-G, Abbott Park, IL, USA). A second small blood capillary sample (~1 mL) was collected to assess baseline blood lactate concentration (La^+^, mmol/L) from the fingertip using a handheld point of care lactate analytic device (Edge Handheld Lactate Analyzer, EDGE USA, Palmdale, CA, USA).

Participants then consumed either 0.3 g/kg of sodium bicarbonate (NaHCO_3_) or a placebo (PL) composed of 5 g of cornstarch, each dissolved in 300 mL of an isocaloric, flavored beverage (Gatorade G2, PepsiCo, New York, NY, USA) served at room temperature. The cornstarch placebo was selected for its inert, non-buffering properties and its comparable viscosity and mouthfeel to the NaHCO_3_ solution. Both beverages were visually identical—opaque and color-matched—to preserve blinding. Each solution was ingested over a 5 min period, with the ingestion time marked as t = 0.

Participants then rested until 15 min prior to their individualized time-to-peak alkalosis (previously determined in Part 1; ~30–45 min post-ingestion), at which point a follow-up arteriovenous blood sample was taken to reassess blood HCO_3_^−^ and gas parameters. Immediately after, athletes performed a standardized 10 min pool-based warm-up. Following the warm-up, participants exited the pool, towel-dried, and rested for five minutes before providing a second pre-performance blood sample for measurement of HCO_3_^−^, blood gases, and blood lactate concentration.

Participants then entered the pool and completed a standardized 4 min duet routine under simulated competition conditions. Each routine was performed with the same duet partner across both experimental trials to maintain consistency. Performances were evaluated by two certified FINA adjudicators who were blinded to trial conditions and scored routines according to official FINA judging criteria. Performance outcomes were assessed across two domains: execution and propulsion. Execution scoring reflects the precision, synchronization, stability, and aesthetic control of movements, emphasizing technical accuracy and artistry. In contrast, propulsion scoring evaluates the effectiveness and height of thrusts, boosts, and lifts generated through powerful leg and arm actions, highlighting the swimmer’s ability to produce forceful movement in the water [4,5,9]. Upon completion of the routine, participants exited the pool, towel-dried, and a post-exercise arteriovenous blood sample (~1 mL) was collected approximately 5 min after performance to assess blood HCO_3_^−^, blood gases, and lactate concentration. Immediately upon completion of the duet, participants reported their rating of perceived exertion (RPE) for the effort using the Borg Scale (0–20) to evaluate perceptual effort [21].

### 2.2. Statistical Analysis

All statistical analyses were conducted using GraphPad Prism 10. Data are reported as mean ± standard deviation (SD). Assumptions of normality were assessed using the Shapiro–Wilk test and were met across variables. Because the repeated-measures factor included only two levels, the assumption of sphericity did not apply. A two-way repeated measures ANOVA was used to assess the main effects of time (pre vs. post), treatment (sodium bicarbonate vs. placebo), and their interaction. When a significant interaction was observed, Bonferroni-adjusted post hoc comparisons were conducted to evaluate within- and between-condition differences. Assumptions of normality and sphericity were assessed using the Shapiro–Wilk and Mauchly’s tests, respectively. To quantify the magnitude of observed effects, Hedge’s **g** was calculated for pre-to-post changes within each condition and for post-intervention between-group comparisons. Hedge’s **g** was selected over Cohen’s *d* due to the small sample size in Part 2 (*n* = 7), providing a bias-corrected estimate of effect size. Effect sizes were interpreted using conventional thresholds: small (**g** = 0.2), medium (**g** = 0.5), and large (**g** ≥ 0.8). Statistical significance was accepted at an alpha level of *p* < 0.05.

## 3. Results

### 3.1. Part 1: Individual Time to Peak Bicarbonate

**Figure 1:**
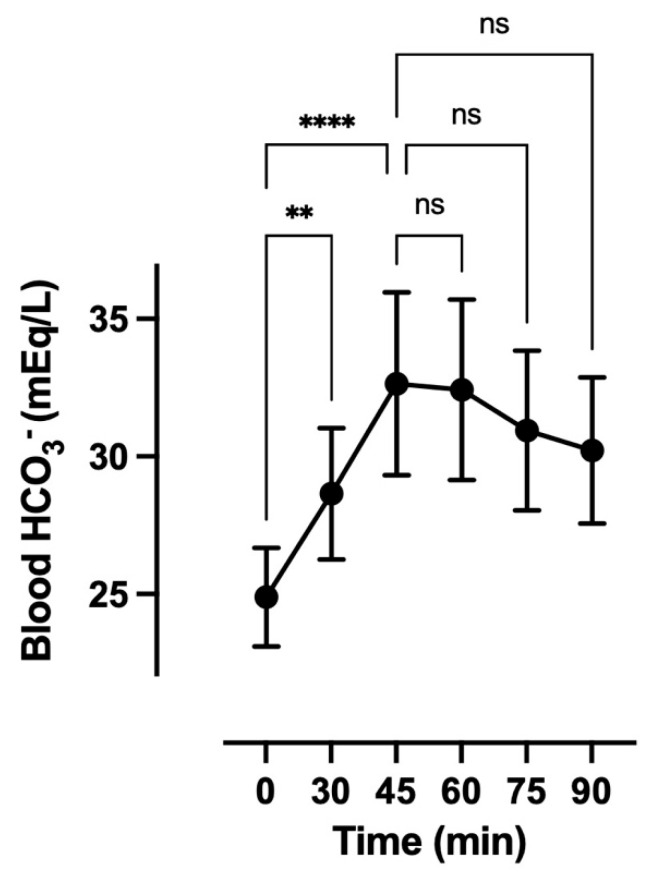
Mean dose–response of 0.3 g per kilogram (g/kg) sodium bicarbonate (NaHCO_3_) on blood bicarbonate (HCO_3_^−^) concentration (mEq/L). Data are mean ± SD. Min, minutes. ** *p* < 0.01. **** *p* < 0.001. ^ns^ mean not significant.

Seventeen elite adolescent artistic swimmers completed individualized bicarbonate profiling to determine the time to peak blood alkalosis following ingestion of 0.3 g/kg body mass of sodium bicarbonate (NaHCO_3_). Peak blood bicarbonate concentration (HCO_3_^−^) was attained between 45 and 60 min post-ingestion, with 53% of participants (*n* = 9) peaking at 45 min and the remaining 47% (*n* = 8) peaking at 60 min, highlighting substantial inter-individual variability despite identical dosing (Figure 1). The average time to peak was 52 ± 9 min. This individualized response corresponded with a mean increase of 6.7 ± 1.8 mmol/L in blood HCO_3_^−^ above baseline values (**g** = 5.03), demonstrating a substantial and consistent systemic alkalosis. These findings emphasize the importance of tailoring NaHCO_3_ timing to individual physiological responses to optimize buffering capacity prior to performance.

### 3.2. Part 2: Effect of NaHCO_3_ on Simulated Duet Performance

#### 3.2.1. Hydration Status

Urine specific gravity (USG) measurements indicated that participants began both experimental trials in a mildly hypohydrated but comparable hydration state. Mean USG values were 1.022 ± 0.002 in the NaHCO_3_ trial and 1.023 ± 0.002 in the placebo trial, suggesting consistent pre-trial hydration across conditions (Table 1).

#### 3.2.2. Blood Acid–Base Status

Blood gas analysis confirmed a significant impact of sodium bicarbonate (NaHCO_3_) supplementation on systemic acid–base balance, both prior to and following performance. Pre-performance blood pH was significantly higher in the NaHCO_3_ trial compared to the placebo (7.46 ± 0.02 vs. 7.37 ± 0.01; *p* = 0.0146; **g** = 5.34), and this alkalotic effect persisted post-performance (7.34 ± 0.02 vs. 7.26 ± 0.03; *p* = 0.0394; **g** = 2.94).

Sodium bicarbonate ingestion also resulted in a marked elevation in blood bicarbonate concentration (HCO_3_^−^), with significantly higher pre-performance values in the NaHCO_3_ condition (29.5 ± 0.9 mmol/L) versus the placebo (22.4 ± 0.4 mmol/L; *p* < 0.001; **g** = 9.55). Although both conditions showed a post-performance decline, the decrease was significantly attenuated in the NaHCO_3_ trial (21.5 ± 1.2 vs. 15.7 ± 1.5 mmol/L; *p* < 0.0002; **g** = 3.99).

Total carbon dioxide (TCO_2_) was similarly elevated in the NaHCO_3_ condition at both time points. Pre-performance TCO_2_ was 30.7 ± 0.9 mmol/L compared to 23.6 ± 0.4 mmol/L in the placebo (*p* < 0.05; **g** = 11.01), and post-performance levels remained higher in the NaHCO_3_ trial (22.6 ± 1.3 vs. 17.7 ± 1.6 mmol/L; **g** = 4.74).

Base excess (BE) further reflected enhanced buffering capacity with NaHCO_3_. Pre-performance BE was significantly greater (5.9 ± 0.6 vs. −2.9 ± 0.5 mmol/L; *p* = 0.0029; **g** = 14.93), and the magnitude of post-performance decline was significantly reduced in the NaHCO_3_ condition (−4.3 ± 0.8 vs. −10.3 ± 1.1 mmol/L; *p* < 0.0001; **g** = 5.84).

These findings confirm that individualized NaHCO_3_ ingestion effectively induces and sustains a robust alkalotic environment before and after high-intensity artistic swimming performance, supporting enhanced extracellular buffering capacity.

#### 3.2.3. Lactate and Perceptual Responses

Post-performance blood lactate concentrations (BLa) were higher in the sodium bicarbonate (NaHCO_3_) condition compared to the placebo (9.3 ± 1.0 vs. 8.4 ± 0.9 mmol/L), indicating a potential increase in glycolytic flux and buffering of hydrogen ion accumulation. While the difference did not reach statistical significance (*p* > 0.05), the effect size was large (**g** = 0.89), suggesting a potentially meaningful physiological impact, which needs to be explored further with more research on this population. In contrast, the rating of perceived exertion (RPE) was significantly lower following the NaHCO_3_ trial (12.9 ± 0.7) compared to the placebo (14.4 ± 0.7; *p* < 0.05, **g** = −2.14), reflecting improved perceptual tolerance to high-intensity effort. These findings support the ergogenic potential of NaHCO_3_ to attenuate perceived fatigue, even under the demanding breath-hold and anaerobic conditions characteristic of artistic swimming.

#### 3.2.4. Performance Outcomes

Judged performance scores indicated a selective improvement in propulsion during the sodium bicarbonate (NaHCO_3_) trial compared to the placebo (6.66 ± 0.20 vs. 6.52 ± 0.16 arbitrary units [AU]), with a large effect size (**g** = 0.85), although this difference did not reach statistical significance (*p* = 0.262). Execution scores remained identical between conditions (6.65 ± 0.15 AU), indicating that technical precision was unaffected by NaHCO_3_ ingestion. These results suggest that acute NaHCO_3_ supplementation may preferentially enhance high-intensity, anaerobically demanding elements of artistic swimming routines—such as propulsion—without detriment to execution-based performance components.

#### 3.2.5. Gastrointestinal Tolerance

Gastrointestinal (GI) discomfort was significantly greater in the NaHCO_3_ condition compared to the placebo at both pre-competition (*p* = 0.022, **g** = −15.34) and post-competition time points (*p* = 0.0056, **g** = −11.31), as shown in Table 2. The highest GI symptom scores occurred following ingestion of the 300 mL NaHCO_3_ solution during the pre-competition period, with a subsequent reduction in symptoms post-performance. The primary contributors to elevated discomfort were reflux, bloating, and nausea; however, these individual symptoms did not reach statistical significance (*p* > 0.05), suggesting a cumulative but variable perceptual effect across participants. Despite elevated GI symptoms, no athletes reported distress severe enough to impair performance or warrant trial withdrawal, indicating overall tolerance within an elite adolescent female athlete population under an individualized ingestion protocol.

## 4. Discussion

This investigation represents the first application of individualized sodium bicarbonate (NaHCO_3_) supplementation in the context of artistic swimming, a sport that uniquely combines prolonged apnea, high-intensity anaerobic effort, and fine motor skill execution. The data demonstrate that NaHCO_3_ ingestion—timed to peak systemic alkalosis—can improve acid–base balance, reduce subjective fatigue, and enhance propulsion performance in adolescent trained female athletes without compromising technical execution or eliciting gastrointestinal distress.

### 4.1. Physiological Mechanisms

NaHCO_3_^−^ acts as an extracellular buffer mitigating the drop in pH caused by hydrogen ion (H^+^) accumulation during anaerobic metabolism. In this study, we observed significantly elevated pre- and post-performance blood bicarbonate (29.5 vs. 22.4 mEq/L pre; 21.5 vs. 15.7 mEq/L post), total CO_2_ (30.7 vs. 23.6 mmol/L^−1^), and base excess (5.9 vs. −2.9 mmol/L^−1^) in the NaHCO_3_ condition. This confirms the efficacy of our dosing strategy in achieving a meaningful metabolic alkalosis. Notably, post-exercise blood pH remained significantly higher in the NaHCO_3_ trial (7.34 vs. 7.26) indicating sustained buffering capacity despite the metabolic acidosis imposed by a maximal routine. An additional consideration is the unique breath-hold physiology of artistic swimming. Approximately half of routine time is performed under apnea, during which rising CO_2_ levels drive respiratory acidosis in parallel with the metabolic acidosis of high-intensity exercise. Sodium bicarbonate may mitigate this dual acid load by augmenting extracellular buffering, thereby facilitating H^+^ efflux during recovery intervals between breath-holds and supporting sustained propulsion while preserving execution. These findings align with systematic reviews and meta-analyses conducted by Carr et al. (2021) and Heibel et al. (2018) which demonstrated moderate effect sizes (Cohen’s d ~0.4) for NaHCO_3_ supplementation on short-duration, high-intensity performance [3,17]. Additionally, individual time-to-peak blood bicarbonate ranged from 45 to 60 min, with approximately half the participants peaking at 45 min and the remainder at 60 min, highlighting substantial inter-individual variability despite identical dosing. This variability is consistent with previous reports of heterogeneous bicarbonate absorption and blood response, which may be influenced by factors such as gastric emptying rate, habitual diet, body mass, and gastrointestinal tolerance. The observed spread of 45–60 min reinforces the importance of individualized timing strategies, as group-mean dosing could risk mistimed ingestion relative to alkalosis onset in nearly half of athletes. Our results add to this body of evidence by confirming benefits in a novel population of trained artistic female swimmers performing sport specific routines.

### 4.2. Higher Lactate Yet Lower Perceived Exertion

Post-exercise lactate concentrations were higher in the NaHCO_3_ condition (9.3 ± 1.0 mmol·L^−1^) compared to the placebo (8.4 ± 0.9 mmol·L^−1^), indicating a greater reliance on anaerobic glycolysis. Notably, this biochemical profile did not correspond with an elevation in perceived exertion. On the contrary, RPE scores were significantly lower in the NaHCO_3_ trial (12.9 vs. 14.4), highlighting a dissociation between traditional biochemical markers of fatigue and subjective effort. Previous work in swimming and rowing has demonstrated that sodium bicarbonate supplementation can enhance buffering capacity and performance outcomes [11,14], though these studies did not specifically assess perceptual responses. Our findings therefore extend the literature by showing that individualized NaHCO_3_ ingestion may confer a perceptual advantage during the high-intensity, apneic efforts of artistic swimming. Such reductions in exertion perception are particularly meaningful in this sport, where athletes must maintain aesthetic expression, synchronization, and technical precision despite extreme physiological strain. By attenuating the sensation of effort, NaHCO_3_ supplementation may help athletes better manage fatigue-induced technical errors or sustain synchronization with partners. While execution scores were unaffected in the present study, larger sample sizes and repeated-event designs (e.g., across a competition weekend) are warranted to more fully evaluate the implications of reduced perceptual fatigue on technical performance outcomes.

### 4.3. Sport-Specific Performance Implications

Although execution scores remained unchanged between conditions, propulsion scores demonstrated a modest improvement following sodium bicarbonate supplementation (6.66 ± 0.16 vs. 6.52 ± 0.20 AU; **g** = 0.85). Although the absolute improvement in propulsion score (0.14 points) may appear modest, this magnitude of change can hold practical significance in competitive artistic swimming, where overall rankings are frequently determined by differences of 0.1–0.3 points between duets or teams. Thus, even a small enhancement in propulsion capacity may provide a meaningful advantage in tightly contested events, supporting the applied relevance of individualized NaHCO_3_ supplementation strategies in this sport. Propulsion elements in artistic swimming require repeated high-power efforts—such as lifts, boosts, and eggbeater kicks—executed under hypoxic and anaerobic conditions [5,6,7,20]. These movement patterns align closely with the physiological demands buffered by NaHCO_3_ supplementation, which has previously shown efficacy in improving high-intensity performance in other intermittent or anaerobic sports. The selective enhancement of propulsion, without compromise to execution, suggests that NaHCO_3_ may preferentially augment performance during the most metabolically demanding components of the routine. Moreover, importantly, no decrement was observed in execution scores, which often depend on fine motor control, synchronization, and postural stability—qualities that could theoretically be disrupted by systemic alkalosis or gastrointestinal discomfort. The absence of any impairment in technical precision supports the safe integration of NaHCO_3_ into artistic swimming routines when individualized timing strategies are applied. Comparisons with other aquatic sports suggest that sodium bicarbonate primarily benefits performance domains with high anaerobic demand, while sport-specific constraints modulate the expression of those benefits. In competitive pool swimming, NaHCO_3_ has repeatedly improved high-intensity interval or time-trial performance, but these efforts are performed largely in normocapnic conditions with only brief underwater phases at starts and turns; by contrast, artistic swimming includes ~50% of routine time under breath-hold, compounding metabolic acidosis with CO_2_-driven respiratory acidosis, which may help explain the selective enhancement of propulsion we observed without changes in execution [8,11,14]. Similarly, individualized NaHCO_3_ timing has improved world-class rowing performance—another high-power aquatic sport performed without prolonged apnea—supporting the interpretation that extracellular buffering preferentially augments short, forceful, anaerobic efforts across aquatic disciplines while leaving fine motor control and aesthetic criteria relatively unaffected.

### 4.4. Gastrointestinal Tolerance and Feasibility in Female Adolescent Athletes

Acute sodium bicarbonate (NaHCO_3_) ingestion is frequently associated with gastrointestinal (GI) distress, particularly when administered as a single bolus. In the present study, GI symptoms were significantly higher in the NaHCO_3_ trial compared to the placebo both pre- and post-performance (pre: 31.58 ± 0.83 vs. 21.00 ± 0.38 AU; post: 25.13 ± 0.59 vs. 19.72 ± 0.23 AU; *p* < 0.05), with reflux, bloating, and nausea being the primary contributors. However, these symptoms were transient, peaking after ingestion and subsiding following exercise. Importantly, no athlete reported symptoms severe enough to impair performance or require withdrawal from the protocol. This tolerance profile may be attributed to the individualized dosing strategy employed. By aligning performance onset with each participant’s time to peak blood alkalosis (mean: 52 ± 9 min post-ingestion), we likely minimized peak GI distress during exertion. These findings are consistent with recent ISSN guidelines recommending personalized NaHCO_3_ ingestion protocols to enhance tolerability [15]. Notably, this study demonstrates that such strategies are feasible and well-tolerated in adolescent female athletes—a population often underrepresented in ergogenic aid research—supporting their safe inclusion in performance optimization practices.

### 4.5. Limitations and Future Research

A key limitation of this study is the small sample size in Part 2 (*n* = 7), which reduced statistical power and increased the risk of type II error, whereby meaningful effects may have gone undetected. While we attempted to mitigate this limitation by using a randomized, double-blind, within-subject crossover design and by reporting Hedge’s **g** to contextualize the magnitude of observed differences, caution is warranted in interpreting the absence of statistical significance for some outcomes. The large effect size observed for propulsion, despite not reaching conventional significance thresholds, highlights the possibility that the study was underpowered to detect performance changes at the level of statistical certainty. Larger-scale studies, across multiple competition formats and athlete populations, are required to confirm and extend these preliminary findings. A second limitation involves the exclusive reliance on subjective, judge-based performance metrics—specifically propulsion and execution scores—which, while ecologically valid, are inherently influenced by human perception and scoring variability. The absence of objective biomechanical or physiological performance measures (e.g., motion tracking, near-infrared spectroscopy (NIRS), electromyography (EMG), or swim velocity analysis) limits the ability to directly quantify the mechanistic impact of sodium bicarbonate on movement efficiency and muscle function. Another limitation is that performance testing was restricted to duet routines. While this provided ecological validity by replicating a standard competition format under controlled conditions, it may limit generalizability to solo or team events. Solo performances place greater physiological demand on a single athlete, whereas team routines introduce additional synchronization and lift elements, both of which may influence how NaHCO_3_ supplementation affects execution and propulsion. Future studies should therefore investigate different routine formats to determine whether the ergogenic benefits observed in duets extend to the unique physiological and technical demands of solo and team events.

Also, while RPE provides important insight into exertional tolerance, it does not directly quantify subjective fatigue. We acknowledge that the inclusion of validated multidimensional fatigue scales would have provided a more comprehensive assessment of perceived fatigue, and this represents a limitation of the current study. Lastly, although some participants showed numerically higher propulsion scores with NaHCO_3_, we did not analyze changes against a smallest worthwhile change (SWC) threshold [22]. Therefore, it cannot be concluded whether these individual responses represent meaningful ergogenic benefits beyond normal variability. Future studies should incorporate SWC-based analyses to better delineate responders from non-responders.

Future research should aim to recruit larger and more diverse athlete samples across multiple performance formats (e.g., solo, duet, team routines) and competitive tiers. Incorporating objective assessments of kinematics, muscle oxygenation, and neuromuscular activation would strengthen the interpretation of buffering effects on performance. In addition, studies investigating chronic NaHCO_3_ supplementation protocols, multi-day competition formats, and co-ingestion strategies with carbohydrates or other buffering agents (e.g., beta-alanine) may provide further insight into optimizing ergogenic effects in sport-specific contexts. As highlighted by recent findings [14], such integrative approaches may offer additive or synergistic benefits, particularly under conditions of repeated high-intensity effort or cumulative fatigue.

## 5. Conclusions

This study shows that individualized sodium bicarbonate (NaHCO_3_) supplementation, timed to peak alkalosis, is a feasible strategy for elite adolescent artistic swimmers. Acute ingestion enhanced systemic buffering, reduced perceived exertion, and selectively improved propulsion under breath-hold conditions, without impairing execution. Although mild gastrointestinal symptoms were reported, they were transient and non-limiting. Given the small sample size and mixed statistical significance, these findings should be interpreted cautiously as preliminary evidence. Practitioners are advised to individualize dosing (~0.3 g·kg^−1^, ~45–60 min pre-performance) with familiarization trials to confirm time-to-peak and tolerance and to consider use when propulsion-heavy elements are emphasized in training or competition.

## Figures and Tables

**Table 1:** Mean experimental trial data for hydration status, perceived effort, blood gas response, and performance scores following ingestion of either a placebo or sodium bicarbonate solution. Data are mean ± standard deviation (SD). * *p* < 0.05 indicates a significant difference between trial conditions (Placebo vs. Sodium Bicarbonate). ^ *p* < 0.05 indicates a significant difference from Pre to Post within the same experimental trial (within-group comparison).

	Placebo Trial		Sodium Bicarbonate Trial			
Measurement	Pre	Post	Pre	Post	*p* Value (Group)	Hedge’s g
USG	1.023 ± 0.002		1.022 ± 0.002		---	---
RPE	---	14.4 ± 0.7	---	12.9 ± 0.7 *	0.045	2.14
Blood Glucose (mmol/L)	5.4 ± 0.2	6.4 ± 0.2	5.9 ± 0.3	6.6 ± 0.3	---	---
Blood pH	7.37 ± 0.01	7.26 ± 0.03 ^	7.46 ± 0.02 *	7.34 ± 0.02 * ^	Pre: 0.015; Post: 0.039	1.2
Blood TCO_2_ (g)	23.6 ± 0.4	17.7 ± 1.6	30.7 ± 0.9 *	22.6 ± 1.3 * ^	Pre: 0.0128; Post: 0.0213	1.4
Base Excess	−2.9 ± 0.5	−10.3 ± 1.1	5.9 ± 0.6 *	−4.3 ± 0.8 *	Pre: 0.0029; Post: <0.0001	2.1
Blood pCO_2_ (mmHg)	38.6 ± 0.8	36.4 ± 1.5	40.8 ± 1.2	39.3 ± 1.2	---	---
Blood HCO_3_^−^ (mEq/L)	22.4 ± 0.4	15.7 ± 1.5	29.5 ± 0.9 *	21.5 ± 1.2 * ^	Pre: 0.0147; Post: 0.0272	1.5
Blood La^+^ (mmol/L)	2.4 ± 0.2	8.4 ±0.9 ^	2.5 ± 0.2	9.3 ± 1.0 ^	>0.05	0.89
Performance Execution	---	6.65 ± 0.15	---	6.65 ± 0.16	0.972	---
Performance Propulsion		6.52 ± 0.20		6.66 ± 0.16	0.262	0.85

USG, urine specific gravity; RPE, rating of perceived exertion; mmol/L, millimoles per liter; TCO_2_, total carbon dioxide; g, grams; mmHg, millimeters of mercury; HCO_3_^−^, bicarbonate; mEq/L, milliequivalents per liter; La^+^, lactate.

**Table 2:** Mean gastrointestinal comfort symptoms (*n* = 7, Part 2) 15 min after ingesting (Pre-Exercise) the placebo or NaHCO_3_ solution and after (Post-Exercise) the competition duet routine. * *p* < 0.05 between the placebo versus sodium bicarbonate trial Pre-Exercise and Post-Exercise. Data are mean ± standard deviation (SD).

	Placebo Trial				Sodium Bicarbonate Trial			
	Pre- Exercise	SD	Post- Exercise	SD	Pre- Exercise	SD	Post- Exercise	SD
Reflux	1.00	0.00	1.00	0.00	3.86	2.04	1.14	0.38
Heartburn	1.00	0.00	1.00	0.00	1.86	1.57	1.00	0.00
Belching	1.00	0.00	1.00	0.00	2.00	1.29	1.00	0.00
Bloating	1.29	0.49	1.14	0.38	2.57	2.70	2.57	2.37
Stomach cramps	1.14	0.38	1.00	0.00	1.29	0.76	1.29	0.49
Nausea	1.43	0.79	1.14	0.00	5.86	2.12	2.71	1.80
Vomiting	1.00	0.00	1.00	0.00	1.00	0.00	1.00	0.00
Lower abdominal cramps	1.00	0.00	1.00	0.00	1.43	0.79	1.29	0.49
Flatulence	1.14	0.38	1.00	0.00	1.14	0.38	1.14	0.38
Urge to defecate	1.14	0.38	1.00	0.00	1.71	1.25	1.29	0.49
Side ache/stich	1.00	0.00	1.00	0.00	1.00	0.00	1.00	0.00
Loose stool	1.00	0.00	1.00	0.00	1.00	0.00	1.14	0.38
Diarrhea	1.00	0.00	1.00	0.00	1.00	0.00	1.14	0.38
Intestinal bleeding	1.00	0.00	1.00	0.00	1.00	0.00	1.00	0.00
Dizziness	1.43	1.13	1.29	0.76	1.29	0.49	1.71	1.50
Headache	1.57	0.98	1.29	0.76	1.14	0.38	2.57	1.51
Muscle cramps	1.00	0.00	1.00	0.00	1.00	0.00	1.00	0.00
Urge to urinate	1.86	2.27	1.86	2.27	1.43	1.13	1.14	0.38
Mean	21.00	0.38	19.72	0.23	31.58 *	0.83	25.13 *	0.59

## Data Availability

The data presented in this study are available from the corresponding author upon request due to restrictions related to the protection of personal information.
